# Episodic representation: A mental models account

**DOI:** 10.3389/fpsyg.2022.899371

**Published:** 2022-07-22

**Authors:** Nikola Andonovski

**Affiliations:** Centre for Philosophy of Memory, Université Grenoble Alpes, Grenoble, France

**Keywords:** episodic simulation, mental model, memory, event cognition, structural representation

## Abstract

This paper offers a modeling account of episodic representation. I argue that the episodic system constructs *mental models*: representations that preserve the spatiotemporal structure of represented domains. In prototypical cases, these domains are events: occurrences taken by subjects to have characteristic structures, dynamics and relatively determinate beginnings and ends. Due to their simplicity and manipulability, mental event models can be used in a variety of cognitive contexts: in remembering the personal past, but also in future-oriented and counterfactual imagination. As structural representations, they allow surrogative reasoning, supporting inferences about their constituents which can be used in reasoning about the represented events.

## Introduction

The first two decades of the twenty-first century have brought important developments to the study of episodic memory. Arguably the key development is the discovery of a close processing connection between remembering events from the personal past and imagining ones that could (have) happen(ed) to us. Neuroimaging studies have implicated a “core” brain network, centered on the medial temporal lobes (MTL), that is consistently engaged in both remembering and imagining events (Schacter et al., 2012; Benoit and Schacter, 2015; Hodgetts et al., 2016; Addis, 2018, 2020). Clinical research has provided further evidence for the connection, with episodic amnesiacs shown to have difficulties imagining novel events and scenarios (Klein et al., 2002; Hassabis et al., 2007; Rosenbaum et al., 2009). Behavioral studies have also revealed parallels, such as analogous temporal proximity effects and dependence on the capacity for visual imagery (D’Argembeau and Van der Linden, 2004, 2006). In addition, episodic remembering and imagination appear to have similar developmental trajectories, with the ontogenetic emergence of one ability coinciding closely with that of the other (Szpunar and McDermott, 2008; Coughlin et al., 2014). The evidence has motivated the hypothesis that episodic remembering is dependent on the operations of an integrated cognitive *system*, which supports a variety of related mental activities, and does so in virtue of its relevant—computational and representational—features.

On the dominant view—popular in both psychology (Schacter and Addis, 2007; Addis, 2020) and philosophy (De Brigard, 2014; Michaelian, 2016)—the function of this “episodic system” is best understood in simulationist terms. The system, thought to be subserved by the widely distributed “core” network, constructs *simulations* of events, retrieving and flexibly recombining previously stored episodic elements. The simulated events can, but need not, be situated at specific points in the past or future (De Brigard and Gessell, 2016). Accordingly, the system supports the remembering of personally experienced events, but also the imagination of counterfactual alternatives to them (De Brigard et al., 2013), of possible future events (Schacter et al., 2017) as well as of fictitious events with no determinate spatiotemporal location (Hassabis et al., 2007). These ostensibly dissimilar mental activities are, indeed, often characterized as varieties of *episodic simulation*, and thus as essentially belonging to the same cognitive kind (e.g., Michaelian, 2016; Addis, 2020; Mahr, 2020).

Yet, despite the popularity of the view, there has been no systematic examination of the notion of *episodic simulation*. In their programmatic papers, “constructive simulationists” have cycled through a number of different conceptions of simulation, ranging from “imitative representation of the functioning or process of some event or series of events,” through “re-enactment of sensory-motor states” to “imaginatively placing oneself in [a hypothetical] situation” (Schacter et al., 2008, pp. 41–42). The discussion, moreover, has been plagued by frequent conflation of representational *vehicles* (i.e., what does the representing/simulating) with the *contents* of episodic simulation (i.e., what is represented; prototypically: events). Hence, the episodic system is often characterized as performing “imaginative constructions of hypothetical events or scenarios” (e.g., Buckner et al., 2008; Schacter et al., 2008; Schacter and Addis, 2009). Hassabis and Maguire (2007), similarly, describe the system as one for scene construction, which they define as a “process of mentally generating and maintaining a complex and coherent scene or event” (p. 299). This lack of clarity in the presentation of the theories’ central notions has led to a host of important problems (Cheng et al., 2016; De Brigard and Gessell, 2016; Andonovski, 2019).

Broadly understood, a simulation is a process of generating a representation that in some way resembles what it represents.^1^ Simulations allow their users to exploit such resemblances and gain knowledge about otherwise inaccessible events or processes (Goldman, 2006; Shanton and Goldman, 2010; Weisberg, 2012). Hence, they function as “epistemic devices” (Fisher, 2006; Moulton and Kosslyn, 2009). Yet, there are different ways in which representations may resemble the domains they represent. In the literature, it is common to distinguish between a narrow sense of simulation as *replication* and a broader one of simulation as *modeling* (Heal, 1986; Fisher, 2006; Goldman, 2006). In replication, the simulating process necessarily resembles the simulated (“target”) one at a fine level of grain, mirroring its principles of operation in advancing from state to state. Using the aerodynamics in a wind tunnel to simulate the aerodynamics in open air is a prototypical example (Blachowicz, 1997; Fisher, 2006).^2^ By contrast, in modeling, the resemblance may only be a high-level, abstract one. The simulating process can, yet need *not*, function in accordance with the same principles as the target process does. Many models only generate symbolic descriptions of a process’ inputs, outputs and intervening states. A computer model of airflow patterns need not be governed by the laws of aerodynamics to provide descriptions of processes that *are* (Goldman, 2006).

Episodic simulationists may seem to be committed to a narrow, replication view of simulation. They regularly emphasize the specific form episodic simulations take, from their quasi-perceptual nature to their characteristic phenomenology of “reliving” or “re-experiencing” the past (e.g., Suddendorf and Corballis, 2007; Michaelian, 2016; Addis, 2018, 2020; Mahr, 2020). Indeed, the high degree of resemblance—phenomenological, neural, and functional—between the processes involved in remembering and those involved in the original experiences of remembered events is sometimes taken to constitute “fairly direct evidence for a simulational account of memory” (Shanton and Goldman, 2010, p. 533).^3^ As Andonovski (2019) has argued, however, the replication view doesn’t sit well with the representationalist commitments of most simulation theories. The reason is straightforward. The episodic system is standardly taken to represent not only (past) mental states or experiences but also a variety of “external” events—birthdays, weddings, holiday trips etc. Indeed, on a prominent recent account, such events are the primary objects of episodic remembering (Michaelian and Sant’Anna, 2021). It is relatively clear that the principles governing these events cannot be directly *replicated* by psychological processes.^4^ Yet, if episodic simulation is taken to involve solely the replication of *mental* processes, the key question remains open: how does the system represent events by simulating mental processes associated with them?^5^ On the other hand, if episodic simulation is to be understood as a kind of modeling, then we are owed an account of the class of relevant models. Absent a careful examination of the *kind* of resemblance between episodic representations and their contents, we would have to admit that remembering and imagining are “simulative” only in a very attenuated sense.

This paper offers such an account. I argue that the episodic system constructs *mental models:* representations that preserve the spatiotemporal structure of represented domains (Johnson-Laird, 1983, 2006). In prototypical cases, these domains are *events:* occurrences taken by subjects to have characteristic structures, dynamics and relatively determinate beginnings and ends. Due to their simplicity and manipulability, mental event models can be used in a variety of cognitive contexts: in remembering the personal past but also in future-oriented and counterfactual imagination. For expository purposes, my focus will be on remembering, a process in which the episodic system constructs models that aim to reproduce the relevant structure of events from the personal past. The models are constructed at recall with information from a variety of sources. As structural representations, they allow “surrogative” reasoning, supporting inferences about their constituents which can be used in reasoning about represented events.

The paper is structured as follows. In section “Episodic representation: A mental models account,” I develop the account, motivating it with evidence from psychology and the neurosciences of memory. In section “Mental models: Remembering, imagining and reasoning about events,” I compare it to existing accounts, clarifying the main commitments. I also have a look at other cognitive operations supported by the episodic system, such as counterfactual and future thought, highlighting the role event models play in reasoning.

## Episodic representation: A mental models account

In section “Mental models as structural representations,” I introduce the basic features of mental models, focusing primarily on their structure-preserving character. In section “Structural models: The medial temporal lobe and beyond,” I show that the episodic system has the computational and representational resources to construct structural models of temporally extended events. The focus is on the hippocampal-entorhinal circuit and its role in the representation of, potentially high-dimensional, cognitive domains. In section “Episodic representation,” I bring the evidence together, arguing that the episodic system constructs mental models, which preserve the—prototypically: spatiotemporal—structure of represented events. In section “Episodic representation: From encoding to retrieval,” I briefly describe the processes supporting episodic remembering, moving “from encoding to retrieval.”

### Mental models as structural representations

The idea that thinking involves the construction and manipulation of internal models that “mirror” the structure of reality can be traced back to the work of Peirce ((1931–1938/1958)), Craik (1943), and Tolman (1948). In its contemporary form (Johnson-Laird, 1983, 2006; Johnson-Laird and Byrne, 1991), the theory of *mental models* has become one of the leading theories of human reasoning. Mental models are structural representations with a number of characteristic properties, including simplicity, kinematicity, and locality. In this section, I briefly introduce their main features.

**FIGURE 1:**
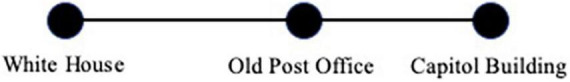
A simple structural model.

Let’s start with the most important feature. Mental models are *structural representations:* they preserve the structure of the domain they represent.^6^ A simple example will allow us to get our feet wet. Suppose that the model in Figure 1 represents the main road connecting the White House and the U.S. Capitol Building. The circles represent buildings in Washington and their relative positions. The model is a structural representation of the road because it preserves (some of) the relevant spatial relations between the buildings, e.g., the *between-ness* relation. Indeed, it is this feature of structural representations that allows for “surrogative reasoning” (Swoyer, 1991): We can learn something about the world by consulting the model.^7^ For example, we can learn that if we take the main road from the White House to the Capitol Building, we are going to pass by the Old Post Office. In our model, space is used to represent space. But that need not always be the case. The relations between the buildings may be represented by relations of a different kind.

In the contemporary idiom, structural representations carry a “second-order structural resemblance” to the domains they represent: a pattern of relations among elements in the represented domain is recapitulated by *some* pattern of relations—spatial, temporal, causal, functional etc.—among elements in the representation (O’Brien and Opie, 2004; Williams and Colling, 2018).^8^ Hence, a *mental* representation may structurally resemble an event as long as it preserves its relevant structure, e.g., via a pattern of functional relations. Second-order resemblance comes in different degrees of strength. While many accounts have focused on isomorphism (a bijective one-to-one mapping for every element in both domains), this is primarily for illustrative purposes (e.g., Cummins, 1996; Shea, 2014). For systems of the kind we are interested in, the resemblance is likely to be much weaker.

This conjures an old problem for resemblance-based accounts of representation. Resemblance is a notoriously undemanding relation, so a theory of representation that aims to be informative owes us some further conditions or constraints (Goodman, 1972; Millikan, 2000). The theory of mental models employs a solution inspired by Peirce’s classic analysis of representation as a triadic relation involving (1) a representational vehicle, (2) a represented domain and an (3) interpretation/use.^9^ A mental vehicle is a structural representation of a given domain just in case the resemblance between the corresponding entities and relations is *used* by the relevant cognitive system.^10^ The system has to be sensitive to the mapping and exploit it for the performance of some downstream operation or task. Structural representations, in other words, rely on their *exploitable similarities* to the domains they represent (Godfrey-Smith, 1996; Shea, 2014). In a recent essay, Gładziejewski and Miłkowski (2017) provide a very useful analysis of this idea, arguing that exploitability should be understood in causal terms. On this view, psychological explanations that invoke structural representations should be construed as causal explanations that feature facts regarding similarity as an explanans and success of failure in the performance of some operation/task as an explanandum (p. 339–345). The structural resemblance between a mental model and a represented domain, that is, needs to be causally relevant to cognitive/behavioral success.

Importantly, mental models are *not* “picture-like” in the sense that every “part” of the model contributes in the same way to determining the model’s content, i.e., by representing a part of what the model represents (cf. Fodor, 2007, 2008). In fact, mental models typically have canonical decompositions and constitutive structures. What the semantic/syntactic constituents of a given model *are*, of course, depends on the way the model is used by the relevant cognitive system. Yet, there is no requirement that every (arbitrarily selected) “part” of the model be a constituent and contribute to the representation in the same way (cf. Burge, 2018).^11^ Relatedly, mental models should not be confused with mental images (see Johnson-Laird, 1996, 2006, Ch. 2). Mental model theorists can accept, but need not be committed to, the existence of mental imagery.

Like most models, mental models are *simplified* representations of target domains. They only contain the information necessary for understanding the relevant structure of a domain, leaving out many (cumbersome) details. This is not only because “fully mirroring” a complex domain may be unachievable, but also because it may not be a good idea. A structural representation needs to be usable by systems with limited computational resources, and the more complex the representation is the less likely it is to be usable. Moreover, simplified models afford a degree of flexibility, allowing systems/agents to use them for representing more than one state of affairs, and in a variety of circumstances. A mental model “can represent what is common to different ways in which a possibility might occur” (Johnson-Laird, 2006, p. 36). A specific exploitable mapping will thus correspond to a *set* of possibilities (e.g., think about all the different ways of “extending” the toy model of Washington above).

Mental models can also be *kinematic*, unfolding in time to represent temporal sequences (Johnson-Laird, 1983). Hence, reasoners can model a sequence of steps necessary to solve a variety of problems, e.g., inferring causal relations from movement of objects in a domain (Khemlani et al., 2014, 2015). Studies have consistently shown that people construct and manipulate such models in a piecemeal, step-by-step way (Radvansky and Zacks, 2014, Ch. 2). Finally, mental models are typically *local:* they represent the structure of a delimited domain (e.g., the domain consisting of the White House, the Old Post Office, the U.S. Capitol building and the road connecting them) without representing the relations to properties of other domains (e.g., the relations between these buildings and the Great Wall of China). The locality of models allows reasoners to explore the structure of a target domain without worrying about its ‘‘global’’ connections.^12^

### Structural models: The medial temporal lobe and beyond

The episodic system is subserved by a distributed brain network, in which the medial temporal lobe and the hippocampal formation play a major role (Schacter et al., 2012; Benoit and Schacter, 2015; Hodgetts et al., 2016; Addis, 2018, 2020). In this section, I focus on the computational interplay between the hippocampus and the entorhinal cortex (EC),^13^ aiming to show that it can undergird the construction of structure-preserving representations of events, i.e., mental event models.

The discovery of *place cells*, neurons that fire preferentially when an organism is in a specific location in its environment (O’Keefe and Dostrovsky, 1971), is a useful starting point. O’Keefe and Nadel (1978) argued that the hippocampus houses an allocentric map of physical space—organized as a Euclidean coordinate space—in which the firing patterns of place cells identify the organism’s location. The story was enriched with the discovery of *grid cells* in the adjacent medial entorhinal cortex (Hafting et al., 2005). Unlike place cells, grid cells fire at multiple (fairly) evenly spaced locations, covering much of the organism’s environment in a grid-like manner. One of the defining features of grid cell firing is their sixfold symmetry: the firing fields surrounding every “node” in the grid form a regular hexagon. Contemporary accounts of (rodent) spatial navigation capitalize on the interplay between these cells, with the activity of hippocampal place cells thought to index locations in a grid-like representation of the environment supported by the medial EC (Bush et al., 2015; Moser et al., 2017). Moreover, we now know that there are other kinds of cells in the MTL that code for additional elements of the map, including head direction cells, landmark cells, distance cells etc. (for this “zoo of cell types,” see Moser et al., 2017).

The maps, purportedly instantiated in the MTL, are *structural* representations of the organisms’ environments in that the “co-activation structure” of the cells is taken, by downstream planning and navigation systems, to recapitulate relevant spatial (topological) relations in the environments, e.g., relative position (Dragoi and Tonegawa, 2013; Bellmund et al., 2018). The evidence that representations generated by this hippocampal-entorhinal system play an important role in motor planning and navigation has slowly accumulated (Moser et al., 2017).^14^ Indeed, this is not in rodents only. Direct recordings of neuronal activity in neurosurgical patients navigating virtual environments have established the existence of place cells (Ekstrom et al., 2003) and EC cells exhibiting grid-like patterns in humans (Jacobs et al., 2013). Moreover, grid coding has also been observed in neural recording at other scales, e.g., in fMRI studies of virtual or imagined navigation (Bellmund et al., 2016; Julian et al., 2018).

Yet, that is not the whole story. The hippocampal-entorhinal circuit, it is now clear, codes for dimensions other than space. First, a number of studies have revealed *temporally* organized MTL patterns involved in memory processing, providing support to the long-held view that the region is critical for memory. A key development is the discovery of so-called “time cells”: cells in the hippocampus and the medial EC that fire preferentially when an animal is at a particular moment in a temporally structured experience (MacDonald et al., 2011; Kraus et al., 2015).^15^ Sequences of time cell activity have been shown to predict accurate memory judgments about specific events (Eichenbaum and Cohen, 2014). Importantly, there is an extensive overlap between the respective populations of time cells and place and grid cells in the hippocampus and the EC. In the provocative words of Howard Eichenbaum: “Time cells are the same neurons as place [and grid] cells” (2014, p. 738). Second, hippocampal cells have been shown to exhibit place-like firing fields even in non-spatiotemporal tasks. Aronov et al. (2017), for example, recorded the hippocampal-entorhinal activity in rats tasked with using a joystick to manipulate sound along a continuous frequency axis. They found a neural representation “of the entire behavioral task, including activity that formed discrete firing fields at particular sound frequencies” (2017, p. 719). The cells involved in the representation overlapped with known place and grid cells in the circuit.

Third, grid-like neural coding has also been found in non-spatiotemporal tasks. Constantinescu et al. (2016) trained participants to associate different symbolic cues with figures of birds, which varied in two independent dimensions: neck length and leg length. This allowed for a representation of possible bird figures in a two-dimensional “bird space,” spanned by these features. While in the fMRI scanner, participants watched short videos of birds morphing in accordance with predetermined neck:legs ratios and were asked to imagine the outcomes had the birds continued to morph with the same ratios. (They were effectively tested on their knowledge of “directions” in the abstract “bird space”). Analyzing the fMRI data, the authors found hexadirectional signals, characteristic of grid coding, in the entorhinal cortex and the distributed (“core”) network associated with episodic thought. In a similar study, Bao et al. (2019) trained participants to navigate a two-dimensional “odor space” and to form predictions about to-be-encountered smells in a natural environment. They also found the distinctive hexadirectional coding in the entorhinal cortex as well as the ventromedial prefrontal cortex. Importantly, the strength of grid coding in the entorhinal cortex correlated with the subjects’ behavioral performance, suggesting that “success on the task requires access to an internalized map of odor space” (p. 1067).

Results of this kind have spurred the development of new theoretical accounts of the role of grid coding in cognition. In a prominent proposal, Bellmund et al. (2018) argue that the hippocampal-entorhinal system can represent experiences in *high*-dimensional ‘‘cognitive spaces.’’ A cognitive space is structured by a set of quality dimensions, which may be tied to sensory but also more abstract, conceptual features. A given stimulus is located in such a space in accordance with its feature values along the relevant dimensions. Importantly for our purposes, the dimensions have underlying metrics and satisfy geometric constraints, such as between-ness and equidistance.^16^ The existence of these constraints makes surrogative reasoning possible, allowing inferences about previously unexperienced stimuli (e.g., if I “move” in *this* direction, I should expect *x*). Bellmund et al. (2020) suggest that the coding principles of the hippocampal-entorhinal circuit are particularly well suited to support ‘‘navigation’’ in such high-dimensional cognitive spaces, with place cell activity indexing locations in spaces mapped by the entorhinal grid system. The underlying representations will be structure-preserving in that distances between positions in cognitive spaces will be reflected in distances between respective vectors of place cell activity. This structural resemblance can be exploited by a number of downstream processes.^17^

There is one final element that needs to be highlighted here: the *remapping* and task-sensitivity of hippocampal cells. A number of studies with rodents have shown that ensembles of place cells can suddenly and collectively alter their firing patterns (i.e., “remap”) in response to relevant environmental cues, e.g., when made to run in an opposite direction on a linear track (e.g., Markus et al., 1995; Redish et al., 2000). Place cells remapping also occurs with changes in goals. For example, when a rat navigates toward a location in a T-maze, different cells fire at the same location depending on whether its goal is on the right or left side of the maze (e.g., Lee et al., 2006). In the human study mentioned above (Ekstrom et al., 2003), the place cell firing patterns change depending on the location one is searching for in virtual reality. Time cells remap in similar ways (Eichenbaum and Cohen, 2014). Hence, in a delayed matching-to-sample task with head-fixed rats, the same cells were involved in distinct temporal sequences associated with different sensory events (MacDonald et al., 2013). These results indicate that hippocampal representations code for features of specific contexts or domains, which may be “segmented” in accordance with the relevant task-and goal- requirements. It is thus important to note that hippocampal activity is modulated by both spatial and *event* boundaries (Ezzyat and Davachi, 2014; Baldassano et al., 2017).

In sum, the evidence suggests that the hippocampal-EC circuit can maintain structure-preserving representations of multiple—potentially high-dimensional—domains, which may be used for a variety of cognitive activities. In the next section, I argue that the episodic system constructs structure-preserving representations of events, employed in remembering the personal past and in various forms of imagination.

### Episodic representation

The episodic system, I propose, constructs mental models of events. The models are local structural representations, replicating the (spatiotemporal) structure of delimited domains; prototypically—segments of time taken to have relatively determinate beginnings and ends (Zacks and Tversky, 2001). The delimitation of the domains is goal- and context- dependent and utilizes event segmentation information as well as semanticized event schemas. Kinematic event models can represent the “micro-time” of events at multiple temporal scales. They will often be chained in sequences, affording the exploration of complex trajectories through event/cognitive spaces. Importantly, the structural resemblance between models and represented events is causally relevant to the performance of the episodic system’s operations and ultimately to the organisms’ cognitive and behavioral success. In remembering, the system constructs event models that aim to reproduce the structure of *past* (personal) events. Due to their simplicity and manipulability, the models can represent distinct ways in which events may unfold. In this section, I examine the locality, structural character and causal relevance of event models in the context of memory. I come back to other representational uses in section “Modeling and surrogative reasoning.”

Consider locality first. The episodic system represents events as relatively disconnected ‘‘micro-domains.’’^18^ Events are important units of human cognition.^19^ People—systematically and automatically—carve the continuous flow of experience into discrete events, organized at multiple temporal scales (Newtson, 1973, 1976; Zacks et al., 2001; Radvansky and Zacks, 2014; Zacks, 2020). Importantly, perceptual event segmentation has downstream effects on memory encoding, organization and recall. Information presented at event boundaries is typically remembered very well (Newtson and Engquist, 1976; Boltz, 1992; Schwan et al., 2000). Moreover, people have been shown to remember information within event boundaries better than they remember information across them (Ezzyat and Davachi, 2011; DuBrow and Davachi, 2013; Pettijohn et al., 2016). During recall, stored information about boundaries—which may be organized in a hierarchical manner (Estes, 1985; Farrell, 2012; Collin et al., 2017)—is used to delimit the relevant domains (i.e., the events to be modeled). Yet, this is only one source of pertinent information. The system also relies on general knowledge, including event schemas and scripts: representations that encode knowledge about what typically happens in events of a particular kind (Schank, 1982; Hard et al., 2006). What information is used, and how it is put to use, depends on the details of the context as well as the goals of the subject (Andonovski, 2021). As we have seen, context and goal structure can modulate the activity of ensembles of cells, enabling remapping. Nevertheless, it is important to emphasize that this is only one component in a widely distributed modeling system. Event-specific patterns of neural activity during remembering can be found in large regions of the cortex (Chen et al., 2017).

Event models preserve the relevant *spatiotemporal* dynamics of represented events. In memories of past events, the represented entities and actions are bound to a spatial context, e.g., < we saw a historical re-enactment in front of the US Capitol Building >. This idea, which goes back to Tulving’s (1983) early work,^20^ has recently led to investigations of the way in which such binding is represented by the episodic system (e.g., Hassabis and Maguire, 2009; Schacter et al., 2012). A key feature of episodic representation, I propose here, is second-order structural resemblance. Event models represent the spatial context of an event by relying on structure-preserving mappings between patterns of spatial relations in the event and corresponding patterns of neural activity. Above, I showed that the hippocampal-entorhinal circuit, which is essential for recalling the spatial context of memories (Eichenbaum and Cohen, 2014), can support the construction of allocentric event models. While the mappings will necessarily be partial, i.e., only *some* relations will be represented-this will often be sufficient to replicate the relevant spatial structure of an event. The evidence suggests that there is a constant interplay between allocentric and egocentric coding in the medical temporal lobes.^21^ Not only does the place-grid system rely on input from egocentrically coded sensory representations, but its allocentric representations are regularly transformed into an egocentric code for the purposes of action, motor planning and indeed memory (Kunz et al., 2020; Rolls, 2020; Wang et al., 2020).^22^

The existence of structural resemblance between patterns of MTL activity and spatiotemporal relations in memory is nicely illustrated by Deuker et al. (2016), who studied the formation of such relations in a large-scale virtual reality setting. Participants in the study had to learn the spatial and temporal relationships between elements placed along a specific route in the virtual environment. The fMRI data showed that subject-specific neural similarity in the hippocampus scaled with the remembered proximity of elements in both space and time. In a recent study, Bellmund et al. (2020) went further, providing a compelling demonstration that the structure-preserving mappings are *exploited* by downstream memory processes. Capitalizing on the fact that trapezoidal boundary geometry distorts the regular firing patterns of entorhinal grid cells, the experimenters used a virtual reality system to design a unique trapezoidal environment and test the impact of EC grid cell activity on human spatial memory. They found the participants’ memory for positions and distances to be significantly degraded. Critically, the participants’ distance estimates were biased in a *systematic* way, showing that they were relying on distorted models—“memory maps”—of the environment. The decrease in their behavioral success, thus, correlated with the decrease in structural resemblance between their models and the environment. This, as Gładziejewski and Miłkowski (2017) point out, is a hallmark feature of structural models.^23^

Mental event models can also preserve the *temporal* structure of represented events. Event models are kinematic, “unfolding” in time to represent the temporal relations linking entities, actions or events. While kinematic models have long played a role in the theory (Johnson-Laird, 1983), the discovery of temporally organized patterns in the MTL provides an important clue about their construction. Two main features warrant highlighting here. First, there is an exploitable mapping between the temporal order in which an event unfolds and the patterns of activity of cell populations firing preferentially at successive moments during the experience of such an event. The sequential activity of these cells, as we have seen, “mirrors” the temporal order of events in a sequence, preserving before-after relations. While there a number of theoretical models of temporal order representation in the MTL, almost all of them capitalize on the structure-preserving mappings between patterns of sequential cell activity and the temporal structures of events (Eichenbaum and Cohen, 2014; Ranganath and Hsieh, 2016; Clewett and Davachi, 2017). Second, these patterns seem to play an important role in the binding of elements within a single episode. Above, we saw that sequences of cell activity predict accurate memory judgments about specific events in rodents. In a recent study, Umbach et al. (2020) reached the same result with human epilepsy patients performing a free recall episodic memory task. Using intercranial microelectrode recordings, they found that the activity of hippocampal and EC neurons, showing the characteristic time cell firing signature, predicted the patients’ judgments about the temporal organization of distinct memories. *How* sequential activity contributes to binding is still an open question, yet it is slowly becoming clear that MTL firing sequences are linked with specific temporally organized memories. At recall, the “replay” of these sequences, driven by a variety of associative mechanisms, facilitates the reconstruction of an event’s structure (Schapiro et al., 2017; Liu et al., 2019). In simplest terms, recalling an individual element from an event tends to cue the recalling of other (subsequent) elements. In this way, the step-by-step construction of kinematic representations recapitulates the temporal dynamics of represented events.^24^ Recent behavioral evidence suggests that structural features of experienced events, including the number and nature of elements (“sub-events”), modulate the recollection of their temporal unfolding, affecting both retrospective and prospective duration judgments (Faber and Gennari, 2015, 2017).^25^

Again, it is vital to underscore that this replay mechanism provides only one, albeit important, component in a widely distributed episodic system. Modeling the dynamics of events, notably, involves a complex interplay between mechanisms in the medial temporal lobe and the medial prefrontal cortex (Barker et al., 2017; Chao et al., 2020). These mechanisms support both the construction of kinematic models, including the integration of sensory and conceptual elements in a spatiotemporal context, and their targeted manipulation. Hence, rememberers can construct *multi-step* models, chaining event models in sequences to represent more complex episodes. In the process, event segmentation information will often be combined with semantic knowledge to represent the structure of complex events at different temporal scales. In some cases, the models will represent nested hierarchies of temporally extended events (Hirtle and Jonides, 1985; Farrell, 2012). Recent studies employing novel representational analysis techniques demonstrate that hippocampal mechanisms do support the construction of such dynamic hierarchical structures, employed in episodic memory and a number of other cognitive activities (Collin et al., 2015, 2017).

**FIGURE 2:**
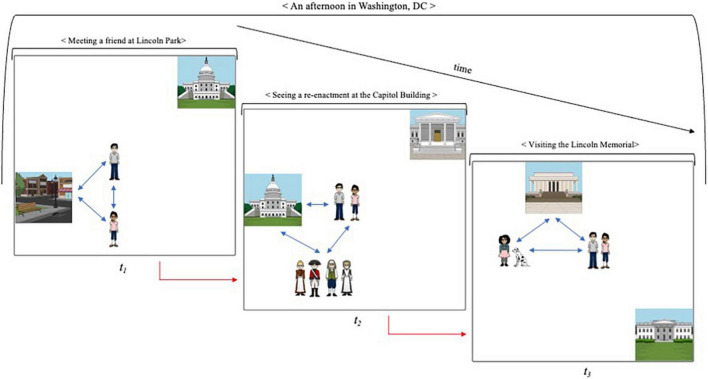
Running a kinematic model of a complex episode. The model simulates the changing states in an episode, re-enacting its spatiotemporal dynamics. As the model “unfolds,” the reconstructed context provides cues for the retrieval of elements associatively bound to it. The blue arrows represent binding of items and locations in a given (sub-)event. The red arrows represent sequential binding of elements in the event sequence.

For expository purposes, I have treated the modeling of spatial and temporal relations separately. Yet, it is the interaction between the two that is of key importance. “Running” a kinematic mental model (Johnson-Laird, 1983; Hegarty, 2004) affords the simulation of the *changing* states of the represented entities and the relevant relations among them. Figure 2 provides a simplified sketch. The model represents a complex memory episode (< An afternoon in Washington, DC >), segmented into three, temporally organized, sub-events. As the episode unfolds, the spatial context of the memory—the represented entities and their relative position—is gradually altered. The model simulates the relevant changes, re-enacting the spatiotemporal dynamics of the episode. (Unlike episodic models, the model depicted in Figure 2 is, of course, *not* genuinely kinematic since temporal relations are represented statically as a sequence of events). At each step of the simulation, the reconstructed context provides associative cues for the retrieval of elements bound to it (cf. Howard and Kahana, 2002; Eichenbaum and Cohen, 2014). In our example, the modeling of the Capitol Building cues both constituent details (e.g., architectural features, people present) and subsequent elements of the event-sequence (“*after that*, we moved in the direction of the Lincoln Memorial”). In that sense, kinematic models aim to capitalize on a well-known feature of memory: matching the environmental context of encoding at retrieval improves recall (Tulving and Thomson, 1973; Smith et al., 1978). Going back to Washington may provide an optimal opportunity for context reinstatement, but it is quite costly and often unfeasible. Running a kinematic model of the event provides an inexpensive, and regularly accessible, surrogate.

It is in this context that the theorized role of the hippocampal-EC circuit in the representation of *high*-dimensional cognitive spaces becomes particularly interesting. The evidence presented above suggests that the circuit codes for features other than space and time, and that its functioning is modulated by task- and goal- relevance. It is thus tempting to hypothesize that it can undergird the construction of models that preserve the structure of events across *multiple*, cognitively or behaviorally relevant, quality dimensions. The dimensions may be tied to sensory (sounds, odors), cognitive (emotions, goals) or even abstract features of events (e.g., dimensions in the “reward structure” of an event/environment). A high-dimensional model will afford a more extensive reinstatement of an event’s context; a high-fidelity surrogate. “Running” the model, the rememberer can chart—and *explore—*complex trajectories in an event’s multidimensional structure. To combine our example with the experimental results, a rememberer may simulate their “movements” not only through the spatial landscape of Washington, but also through a gradually changing sound or odor context—from the smell of freshly cut grass on the White House lawn to the hush in the stuffy Library of Congress. This proposal provides a new angle on the familiar idea that remembering a past event comes with a sense of “re-living” or “re-experiencing” it. This may be not only because of the retrieval of modality-specific sensory information but also because remembering involves the construction of models that reproduce the event’s *multi*-dimensional structure (or, at least, its relevant components). While this proposal is admittedly speculative, emerging behavioral and neural evidence suggests that it presents a promising direction for investigation (cf. Ekstrom and Ranganath, 2018).

I close this section by linking the proposal to the familiar representational deficits of subjects with hippocampal/MTL damage. The most well-documented are the deficits in the generation of *spatially* coherent (“integrated”) representations of events or scenes (Hassabis et al., 2007; Rosenbaum et al., 2009).^26^ Hassabis et al. (2007) tested amnesic patients with bilateral hippocampal damage on their capacity to imagine new experiences in response to short verbal cues, e.g., “imagine lying on a sandy beach in a beautiful tropical bay.” They then used a variant of the Autobiographical Interview (Levine et al., 2002) to measure the richness of the representations on the basis of several distinct components. The component that matters most for our purposes is the “spatial coherence index,” calculated from the participants’ responses to a set of statements describing their experiences. Some of these statements indicated that aspects of the representation were integrated (e.g., “I could see the whole scene in my mind’s eye”), while others indicated fragmentation (e.g., “It was a collection of separate images”). The scores of hippocampal amnesiacs were significantly lower than those of controls, indicating a marked impairment in the generation of a spatially coherent/integrated representations of events. This was despite the fact that amnesiacs could represent, and reason about, the spatial relations between pertinent elements (in a “semantic” way), a result familiar from the existent literature (Rosenbaum et al., 2009).

A careful analysis of the evidence—from Hassabis et al. (2007) and subsequent studies—suggests that the fragmentary character of event representations in individuals with MTL impairments may not be limited to space. Indeed, they seem to struggle constructing integrated *kinematic* representations. A striking illustration of this struggle is provided by Susie McKinnon, one of the participants in a study investigating deficits of retrieval linked to impaired activation of midline cortical structures (Palombo et al., 2015). When McKinnon attempts to remember/imagine being at the beach, she runs into difficulties:

[I can] visualize a hammock, maybe. And then there’s probably a palm tree. As soon as, in my mind, I’d try to grab that palm tree, I lose the hammock. I can’t hold in my mind more than one move ahead (Hayasaki, 2016).

These difficulties affect McKinnon’s ability to structurally model temporally ordered events or sequences of experiences. If she tries to remember walking on the beach, for example, she cannot bind the constituent elements in a representation that reproduces the temporal dynamics of the event. [In her words: she can’t hold the “moves” in her mind, an impairment which affects her ability to perform a variety of activities, including playing chess (Hayasaki, 2016)]. This report is rather typical. As De Brigard and Gessell (2016) point out, many of the existing experimental results can be accounted for by the hypothesis that individuals with MTL damage have difficulties constructing dynamic representations, with components that unfold over time (e.g., Rosenbaum et al., 2009; Romero and Moscovitch, 2012; Race et al., 2013). Additional support for this claim comes from studies investigating the problems such individuals have with the reinstatement of the temporal context of memories (Palombo et al., 2019). Relatedly, patients with MTL epilepsy show significant impairments in remembering/reconstructing the moment-by-moment “unraveling” of a past episode (e.g., St-Laurent et al., 2011). Individuals with MTL impairments, I conclude, have difficulties constructing kinematic models that reproduce the patterns of spatiotemporal relations in represented events. This may be because of impairments in mapping mechanisms or, perhaps more likely, because of problems with the downstream exploitation of structural resemblances in memory or imagination tasks. If I am correct about the relevance of context reinstatement is on the right track, these deficits may also lead to additional problems with event detail generation (Rosenbaum et al., 2009; St-Laurent et al., 2009, 2011).

In this section, I argued that the episodic system constructs mental models of temporally extended events, used in remembering and imagination. In the next section, I briefly describe some processes that support the construction of such models in the context of memory—from encoding to retrieval.

### Episodic representation: From encoding to retrieval

#### Encoding

What information gets encoded upon experience of an event depends on a variety of factors—goals, context, resources etc. It is relatively uncontroversial, however, that much *can* be encoded, even when the events are comparatively simple. Most obviously, there is information about the perceived event structure. This includes details about the pertinent entities and properties as well as the relations among them (Aggleton and Brown, 1999; Joensen et al., 2020). The evidence surveyed above suggest that information about event boundaries is also routinely encoded. This may be in the form of discrete, hierarchically organized, “episode markers” that represent the beginnings and ends of different events (Khemlani et al., 2015). Moreover, there is now a lot of evidence that information from multiple stages of stimulus processing can be encoded and affect subsequent behavior. Much of it comes from the transfer-appropriate processing (TAP) tradition (Morris et al., 1977; Bransford et al., 1979), the key tenet of which is that memory of a stimulus is affected not only by the depth of processing, but also by the specific processes/operations performed at encoding.^27^ This is well illustrated by a study by Payne and Baguley (2006), who had participants read descriptions of situations, only some of which described integrated spatial configurations. (Thus, only those descriptions supported the construction of a coherent mental model.) In a surprise recognition test, reordering the sentences significantly affected the participants’ memory for the situations, but *only* for the integrated spatial descriptions. Reordering, the authors reasoned, doesn’t affect the content of the final representation, but it does affect the process of constructing a model of the situation. Hence, the results suggest that a *trace* of that construction process in encoded and retained. The participants’ memory was affected because reordering reduced the similarity between processing at encoding and retrieval. This idea fits nicely with the well-documented finding that memory for events reflects *how* people thought about them during encoding.

#### Consolidation and storage

While much can be said about consolidation and storage, here I’ll highlight a single issue—the construction of *event schemas.* On the standard accounts, event schemas are semantic representations that encode knowledge about event types, including elements, properties and relations typical of events of a given type (Schank, 1982; Hard et al., 2006). To construct an event schema, a system must (a) “recognize” that events share important structural similarities, (b) abstract the relevant structural knowledge, and (c) organize it to make applications to, and inferences about, new situations possible. What is particularly interesting is that the mechanisms sketched in the previous sections provide the basic computational resources for the construction. This is illustrated by Whittington et al. (2019), who re-cast both spatial and relational memory problems as instances of structural abstraction. They show that the computational division of labor between different kinds of cells in the hippocampal-EC circuit make such abstraction possible. The details are beyond our scope, but the idea is straightforward. The circuit supports factorization of representations, with unique elements of an event represented separately from its relational structure. These representations can subsequently be re-combined to form a model of a novel event, an idea familiar from the debates about episodic memory. Hence, factorization facilitates abstraction of structural knowledge about event-types, embedded in a model—for our purposes: event schema—that supports application to novel circumstances. In consolidation, the schema is updated and streamlined for optimal inference.

#### Retrieval

**FIGURE 3:**
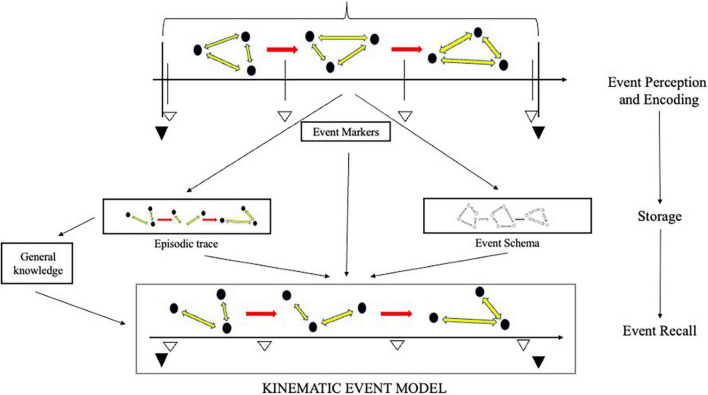
Event model construction in episodic recall. A kinematic event model is constructed at recall with information from a variety of sources, including episodic traces, event markers, and schemas. How much weight is given to information from the different sources depends on a number of factors; most notably—availability, goals and context. The construction of an event model itself leaves “procedural” traces that can be exploited in subsequent recall.

At retrieval, the context and intentions of the subject regulate the activity of the episodic system. The system may, thus, “aim” to construct a model for the purpose of remembering a unique past event, but also for remembering repeated events or imagining possible ones. Just *how* this “aim,” and consequent activity, are determined is very much an open question. Provisionally, we can work with Michaelian’s (2016) proposal, taking the talk of “aim” to be a shorthand for some complex story about the system’s responses to a variety of retrieval cues, provided by the subject or environment. Depending on the aim, information from different sources will be used in the construction of the relevant model. Yet, even in remembering singular past events, the system will rely on a multiplicity of sources. These may include discrete episodic traces as well as semantic information. The construction of an event model will, accordingly, involve a complex interplay between the hippocampal-entorhinal circuit and a number of regions across the core network and the wider neocortex (Schacter et al., 2012; Barker et al., 2017). How much weight is given to information from the different sources depends on a number of factors, e.g., availability, context, goals etc. Given the well-documented loss of episodic information during consolidation and storage (Piolino et al., 2009; Petrican et al., 2010; Tompary and Davachi, 2017), however, the semantic “scaffolding” will typically play a major role (Irish, 2016). Figure 3 provides a diagram of event model construction at recall.

## Mental models: Remembering, imagining and reasoning about events

In section “Episodic representation: What the thesis is not,” I clarify the main thesis of the paper, providing important details about its nature and scope. In “Episodic simulation: Polishing the common core,” I examine the proposed account in the context of the most prominent simulationist accounts in the literature. In “Modeling and surrogative reasoning,” I highlight the role models play in surrogative reasoning about events, examining their use in episodic counterfactual and future thinking.

### Episodic representation: What the thesis is not

I have argued that the episodic system constructs mental models of events: representations that carry second-order structural resemblance to represented events. The models are employed in remembering the past and in various forms of imagination. By detailing their formation and structure, the account aims to characterize the causal contribution the episodic system makes to these cognitive activities. In this sense, the thesis of the paper concerns the *causal-role function* of the system (Cummins, 1975, 1983; De Brigard, 2014). There are, however, a number of related theses about the episodic system and its involvement in remembering and imagination, some of which very prominent in the literature. In this section, I briefly discuss six important ones, clarifying the main commitments of the account.

First, the thesis concerns the content and format of representations produced by the episodic system, *not* the content of episodic memory *experiences.* In the philosophical literature, it is standard to characterize recollective experiences as contentful states, with a number of influential theories of episodic memory content on offer (Dokic, 2014; Mahr and Csibra, 2018; Fernández, 2019). While I have argued that structural event models are employed in remembering, this does not entail that the content of memory experiences is fully determined by the content of these models (cf. Pan, 2022).^28^ A number of different systems—including memory, mindreading, and metacognitive systems—may causally contribute to the content of such experiences. The mental models account is thus compatible with views that posit *richer* episodic memory content, including an autonoetic attitude (Mahr and Csibra, 2018), causal self-reference (Fernández, 2019) or a sense of pastness (Boyle, 2020). Since mental models *are* employed in remembering, the endorsement of such a view nevertheless requires an additional functional account of the models’ integration with representations from different systems. (How specifically is model content “enriched” with the extra elements characteristic of remembering?) Mahr’s (2020) recent proposal of a propositional scope syntax, regulating the use of episodic representations, is a good template for future work.

Second, the mental models account does *not* posit *constitutive* conditions for episodic memory. Simulationists typically characterize remembering as an activity or operation *of* the episodic system—a kind of episodic simulation (Addis, 2020; Mahr, 2020). This characterization is most conspicuous in the work of Michaelian (2016), who considers a mental state/experience to be an episodic memory if and only if it is produced by a properly functioning episodic system “aiming” to represent an event from the subject’s personal past (p. 107). For the reasons highlighted in the previous paragraph, the mental models account is not committed to a view of this kind. While the episodic system may necessarily be involved in the production of memory states/experiences, this does not entail that their “mnemicity” constitutively depends on the operations of the system. Relatedly, the possibility that an appropriate causal connection to a past event *is* a constitutive condition is not excluded. The account is thus compatible, at least in principle, with both causalist (Martin and Deutscher, 1966; Werning, 2020) and anti-causalist (Michaelian, 2016) theories of memory.

Third, the mental models account is *not* a *general* account of memory for events and their properties.^29^ Evidence shows that perceptual event segmentation has downstream effects on long-term memory, which can be manifested in different kinds of recall and a variety of experimental tasks, including recognition memory (Newtson and Engquist, 1976), temporal order and distance memory (Wang and Egner, 2022), and narrative memory (Radvansky et al., 2014). The current account does not aim to characterize the acquisition, storage and organization of information in memory that explains the performance on all these tasks. Rather, its focus is specifically on *episodic* recall for events, with episodicity defined in reference to the computational operations of a particular, functionally integrated, cognitive system. Relatedly, while the account is in principle compatible with different architectural theories of event cognition, their specific commitments may differ. Unlike Franklin et al. (2020) and Kelly et al. (2020), for example, the account is not committed to the distributed representation of stored items in hyperdimensional spaces of the kind posited in vector symbolic architectures. This is despite the employment of similarity spaces, which may constitute a *lingua franca* of cognitive architectures (Lieto et al., 2017). In contrast, the account *is* committed to the claim that episodic models, constructed at recall, do not only represent but functionally recapitulate an event’s structure.

Fourth, the thesis does *not* concern the *evolutionary* function of episodic modeling (Wright, 1973; Millikan, 1984). Simulationists have long discussed the selective advantages of episodic memory and future-oriented imagination (Tulving, 2002; Suddendorf and Corballis, 2007; Schacter, 2012). In recent years, there have been more systematic treatments of the evolutionary function of episodic modeling, re-examining the relationship between remembering and imagining (Jablonka, 2017; Mahr and Csibra, 2018; Boyle, 2019; Schulz and Robins, 2022). These typically employ a kind of “form-to-function” reasoning, inferring the function of the episodic system from features of phenotypic form, i.e., from the structure of the system’s proprietary operations and representations. The mental models account does not offer a view of this kind. By characterizing the *causal*-*role* function of the system, however, it provides ingredients required for “reverse engineering” the design considerations that may have governed its selection (Dennett, 1995).

Fifth, the account is *not* committed to the view that episodic models are *purely* structural. The models are taken to replicate the—paradigmatically: spatiotemporal—structure of represented events. Yet, they may still contain non-structural elements (Johnson-Laird, 2006, Ch. 2). Indeed, a kinematic model of an event will typically incorporate discursive representations of objects (Hebart et al., 2018; Altmann and Ekves, 2019), agents and their properties (Wagner et al., 2012; Bonin et al., 2015), and various non-spatiotemporal relations (Goodwin and Johnson-Laird, 2005; Halford et al., 2010). It will thus have a canonical decomposition and constitutive structure. Hence, an episodic model can be aptly characterized as structural-discursive hybrid (cf. Quilty-Dunn and Mandelbaum, 2019). Yet, as we have seen above, the episodic system has the computational resources to construct models that preserve the structure of events across *multiple* sensory or conceptual dimensions. While there are many open questions concerning the “addition” of dimensions beyond space and time, it is very likely that the process is modulated by task- and goal- relevance (Ekstrom and Ranganath, 2018).

Finally, the account is *not* committed to the view that *memory traces* preserve the structure of events. On a historically influential view, traces are structurally analogous to the events or experiences they represent, affording appropriate recall (Martin and Deutscher, 1966). This view faces a variety of problems, particularly in light of accumulating evidence of content transformation and (re)construction at all stages of memory processing (Schacter et al., 2012; Michaelian, 2016; Andonovski, 2021). As argued in section “Episodic representation: A mental models account,” episodic models are constructed at recall with information from a number of sources, with episodic traces being the most important one. The traces may, but need *not*, be structural analogs of past experiences. All that the account is committed to is that they afford the construction of structural event models at recall. How exactly this occurs is an open question. It does bear emphasizing, however, that recall likely involves not just reactivation of previously stored sequences (cf. Cheng and Werning, 2016; Werning, 2020) but also integration of different kinds of information—sensory and conceptual—into a coherent episode model.

With these qualifications, we can return to the causal-role function of the episodic system. In the next section, I compare the proposed account of this function to the most prominent simulationist accounts in the literature.

### Episodic simulation: Polishing the common core

The mental models account aims to capture much of the common core of the leading theories of episodic simulation. Yet, it also attempts to clarify and systematize some of the main commitments, adjusting boundaries and sharpening concepts along the way. The most ambitious adjustment is integrative—the attempted unification under the umbrella of the theory of mental models (Johnson-Laird, 1983, 2006). The hope is that the umbrella will provide adequate cover, making the traversal through a difficult terrain a little less daunting. Yet, like all umbrellas, this one has a finite span, so some aspects of the theories will be exposed to the elements. The proposal, i.e., is bound to be somewhat revisionist, particularly around the edges. Here, I examine it in the context of the most prominent simulationist accounts of the episodic system, pinpointing the adjustments and revisions.

The main theoretical contribution of the proposed account lies in the analysis and precisification of the notion of episodic simulation. I have argued that episodic simulation is best understood as a process of constructing structural representations that carry second-order resemblance to represented events. On this view, to simulate an event—in the specific sense that the episodic system simulates events—is to construct a kinematic mental model, which aims to recap the event’s (spatiotemporal) structure and dynamics. This modeling notion of episodic simulation, adopted from the literature on perception and reasoning (Johnson-Laird, 2006), seems to fit well with recent versions of the “constructive episodic simulation” hypothesis (e.g., Addis, 2018, 2020; Arzy and Schacter, 2019). Yet, there is another sense of episodic simulation—episodic simulation as the *replication of past mental states or experiences* of events—which has also been prominent in the simulationist literature (see, e.g., Schacter et al., 2008, p. 40–42; Shanton and Goldman, 2010, p. 532–535; Michaelian, 2016, Ch. 7; Addis, 2018, p. 71–73). This second notion of episodic simulation is typically supported by evidence of reactivation, in episodic recall, of content-sensitive patterns of neural activity evoked during the original experience of an event (Danker and Anderson, 2010; Ritchey et al., 2013; Gordon et al., 2014). These patterns are thought to underlie the common impression of “reexperiencing” or “reliving” past events in memory.

The two notions of episodic simulation are often discussed together and are indeed sometimes run together (Michaelian, 2016; see the discussion in Andonovski, 2019). Yet, they characterize different kinds of processes which may, but need not, co-occur in the operations of a cognitive system. Theories of episodic modeling, of the kind defended in this essay, can thus be formulated and defended without appeal to evidence of neural reactivation and/or reinstatement. Given that such evidence does exist, however, the theoretical focus on modeling requires some justification. Three main reasons for favoring a modeling view are worth highlighting. First, the view offers a clear account of episodic *representation* in terms of cognitively exploitable similarities between events and kinematic models of them. Such an account fosters consilience not only with a leading theory of human reasoning but also with theories of structural representation that have grown increasingly popular in the recent literature (Shea, 2014; Gładziejewski and Miłkowski, 2017; Williams and Colling, 2018). The endorsement of a modeling account of episodic simulation, importantly, does not preclude a *causal* role for neural reactivation in episodic recall.^30^ Yet, mere reactivation of patterns of neural activity associated with an experience of an event is not sufficient for event representation. This is not only because the degree of reactivation is often very low (see below), but also because the same neural resources—regions, modules, and indeed individual neurons—are regularly employed in the representation of similar events and features (Danker and Anderson, 2010; Xue, 2018). A generalized replication theory of episodic simulation would have to show how the reactivation of content-sensitive neural patterns is exploited by the system for the representation of “external” events in memory and imagination. Given the structural complexity of typical events, it is plausible that *some* kind of structural modeling will turn out to be an indispensable feature of such an account (cf. Aronowitz, 2019). Second, recent evidence has raised some doubt about the prevalence and importance of neural reactivation in recall. A number of studies have cataloged systematic spatial and temporal transformations of neural patterns from perception to memory in sensory and frontoparietal cortices (Baldassano et al., 2016; Favila et al., 2020; Long and Kuhl, 2021). Indeed, some regions have been shown to respond more strongly to remembered than to perceived events (Favila et al., 2018). Moreover, optogenetic studies have demonstrated that the activation of even a small percentage (2–3%) of cells labeled during learning can lead to context-appropriate behaviors (Liu et al., 2012). These results point to the third reason for favoring a modeling account. The episodic system simulates a wide range of events, for a variety of cognitive tasks. To do so successfully, and in a flexible manner, the system needs to rely both on reinstatement of past neural patterns *and* on systematic processes of transformation of information acquired on different occasions (Stickgold and Walker, 2013; Andonovski, 2021).

These considerations support the characterization of episodic simulation as a process of modeling the structures of represented events. I have criticized “constructive simulationists” for lack of clarity about the notion of simulation, leading to concerns about the theory’s commitments. The merger with structural modeling accounts provides a theoretically robust notion, clarifying the empirical conditions for identifying instances of episodic simulation. Evidence for kinematic modeling should demonstrate the existence of exploitable—and, ideally, experimentally manipulable—similarities between mental representations and events. The exploitability should be manifested in the causal/behavioral relevance of such similarities in spatial memory tasks (as in Deuker et al., 2016; Bellmund et al., 2020) but also in paradigmatic episodic memory and imagination tasks. The modeling account thus provides a good platform for future theoretical and experimental investigation of episodic simulation.

The account also builds on the work of “scene construction” theorists (Hassabis and Maguire, 2007, 2009), aiming to remedy some important conceptual problems. The theory has been criticized for lack of clarity about whether scene construction is to be understood as a hypothesis about the representational vehicles or the contents of episodic representations (De Brigard and Gessell, 2016). As we have seen, this is not a scholastic concern. Individuals with MTL damage can seemingly represent, and think about, complex scenes/events, a finding that doesn’t sit well with the latter interpretation. In addition, the theory has been charged with vagueness about the notion of “space” and “spatial coherence” in scene representation (Ekstrom and Ranganath, 2018). The mental model account improves upon these shortcomings, opting for a divide-and-conquer strategy. The episodic system constructs representations with characteristic content *and* format. The prototypical contents are events—or, indeed, “scenes” from events—represented in a particular way: by models that reproduce their spatiotemporal structure. The unique feature of episodic simulation lies in the structural correspondence between contents and vehicles. From this perspective, it is unsurprising that amnesiacs can still represent aspects of events, including spatial relations. What they cannot do is *mentally model* them, in the sense developed above. In response to the second challenge, the proposed account is linked to the revival (and rebranding) of the theory of cognitive maps. It this tries to lend some clarity to the proposition that spatial coherence is of central importance to episodic representation, while simultaneously exploring the possibility that mechanisms in the MTL can represent other, *non*-spatial, features. Lastly, it puts more emphasis on the kinematicity of event models and its role in the representation of the “micro-time” of events (Hassabis and Maguire, 2009).

The relation to “mental time travel” views of the episodic system is more complicated. This is for a variety of reasons, not the least of which is the absence of a canonical mental time travel account to compare the proposal to. Existent accounts vary both in the interpretation of, and emphasis placed on, different cognitive and phenomenological features (Tulving, 2002, 2005; Suddendorf and Corballis, 2007; Klein, 2015). While this is not the place for a detailed analysis, I will briefly examine two prominent features, seemingly at odds with the mental model view. The first one concerns the experience of “traveling through subjective time—past, present, and future” (Tulving, 2005, p. 9). There are two main reasons for not including this feature in an account of the episodic system. First, emerging evidence suggests that the representation of temporal orientation is independent of episodic modeling (Mahr et al., 2021), with the episodic system also engaged in the representation of events not placed at particular points in time at all (for a review, see De Brigard and Gessell, 2016). Second, despite some important experimental work (e.g., Anelli et al., 2016; Gauthier and van Wassenhove, 2016) as well as a number of interesting theoretical proposals (Arzy et al., 2009; Buonomano, 2017; Thönes and Stocker, 2019), our understanding of “subjective time,” and its role in memory and imagination, is still in its infancy.

The second, closely connected, feature concerns the role of the *self* in episodic memory and imagination. This is typically taken to be manifested in the experience of *autonoesis*—the feeling/thought associated with having experienced an event “first-hand” (Tulving, 1985; Mahr and Csibra, 2018)—and in the ways in which elements of the self are “projected” to the past and future (Tulving, 2005; Buckner and Carroll, 2007). The examination of the connection between episodic modeling and self-related processing certainly deserves a more comprehensive treatment. Nevertheless, there are four lines of evidence pointing to their functional separability. First, there are a number of reports of “selfless” memories, not accompanied by autonoetic experiences, in both clinical and extra-clinical contexts (Klein and Nichols, 2012; Gentry, 2021; Millière and Newen, 2022). Second, episodic information has been shown to be “implicitly” retrievable for a variety of cognitive tasks (Sheldon and Moscovitch, 2010; Wimmer and Shohamy, 2012). Third, there is neuropsychological evidence for selective impairments of episodic simulation and self-related processing (Arzy et al., 2009; Andelman et al., 2010). In a notable study, Kurczek et al. (2015) report a double dissociation between patients with bilateral hippocampal damage and patients with bilateral medial prefrontal cortex damage. While patients in the first group were impaired in their ability to construct detailed event simulations, but were able to incorporate themselves in narratives of the events, those in the second group were able to construct detailed simulations, yet incorporated themselves in narratives in much lower frequency compared to healthy participants. Finally, recent neuroimaging evidence suggests the existence of two distinct “subnetworks” associated with episodic modeling and self-related processing, the first centered on the MTL, the second on the ventromedial prefrontal cortex (Andrews-Hanna et al., 2010; Dafni-Merom and Arzy, 2020). Taken together, the evidence points to the functional distinctness of episodic modeling and self-related processing. That said, the modeling account is compatible with the view that the use of kinematic models in remembering and imagination is often, perhaps even in the majority of cases, accompanied with an experience of the self (in “subjective” time).

This section has examined the relation of the mental models account to the most prominent *simulationist* accounts of the episodic system. While there are influential non-simulationist accounts in the literature, a detailed investigation of their commitments, and (dis)similarities to the proposed view, are beyond the scope of this paper. The proposal’s primary goals are to analyze the notion of episodic simulation as kind of structural modeling, systematize the evidence for the existence of such modeling in episodic thought, and consequently explore the prospects for integration with the theory of mental models. If my arguments are on the right track, there is sufficient promise for such integration, allowing us to exploit the theory’s resources in accounting for episodicity phenomena. In the final section, I offer a sketch of some of the more interesting ideas and connections.

### Modeling and surrogative reasoning

By preserving the structure of represented domains, mental models afford “surrogative reasoning” (Swoyer, 1991). They can be used as stand-ins, supporting inferences about its constituents, which can then be transformed back into information about the represented domains (Craik, 1943; Johnson-Laird, 1983, 2006; Johnson-Laird and Byrne, 1991). By consulting and manipulating the models, we can learn something about these domains, and form new beliefs about them. Episodic models are similarly inference-supportive. They allow subjects to make novel inferences about represented events and reason about their constituent elements. This is facilitated by what Aronowitz and Lombrozo (2020) call “representational extraction,” a process in which information is made available to a system in a new way. We already saw this process at work. In constructing event models, the episodic system extracts information from a variety of sources, much of which not directly available for verbal report or reasoning. With the construction of an *integrated* model, its accessibility conditions change (Elga and Rayo, 2021)-information about the constituent structure of represented events is made available for such processing.^31^ Subjects can thus consult the model, “read out” the relevant information and use it in reasoning, past- or future- related. Because of their simplicity and manipulability, models can be used to represent commonalities among different ways in which events of a particular kind can unfold. Hence, they can be used in a number of cognitive contexts. The specifics of the context, and intentions of the subject, will determine a model’s use and the conditions for representational success.

The most elementary form of such surrogative reasoning involves the extraction of information about spatiotemporal relations among constituent elements. Consulting a model of the afternoon in Washington, a subject may notice/infer some such relations they did not pay attention to beforehand. They may, for example, notice that they must have passed by the Lincoln Memorial at a particular time of day or that they did so *before* stopping for dinner. While these may seem like trivial accomplishments, they require a complex series of cognitive operations. Here, I suggest that it is the reconstructive modeling that makes such reasoning about past events possible. More importantly, as a kinematic model unfolds, the simulated context will provide associative cues for both elements contingently bound to it *and* for semantic knowledge about the elements, context and the prototypical structure of relevant event-types. Given the potential depth and variety of such knowledge, the event model may support inferences of indefinite complexity. This should be familiar to us from the legal context, where eyewitness testimonies can serve as “anchors” in the forensic exploration of the structure and vicissitudes of past events [such exploration will often feature flag phrases like “it couldn’t have been” (e.g., longer than 5 min) or “it must have been” (e.g., very close to the hotel where we were staying at)].

Yet, it is the manipulability of episodic models that makes their flexible use possible. Consider counterfactual thought first. The episodic system has been shown to be engaged when participants are asked to think of “alternative” ways in which past events could have transpired (De Brigard and Giovanello, 2012; De Brigard et al., 2013). On the mental model view, people generate counterfactual alternatives by making changes to models of actual situations (Byrne, 1997, 2002; Johnson-Laird and Byrne, 2002).^32^ The counterfactual alternative will often consist of a simple alteration of the layout (e.g., removal or addition of an object). Thinkers will then compare a model of the actual situation (or what they hold to be true) to the *minimally* altered model to produce counterfactual thoughts. Hence, the smaller the deviation from the “factual” model, the more plausible the counterfactual scenario will seem to them. In the episodic context, the plausibility of a counterfactual “event” will depend on how minimally it diverges from the actual event memory. This is precisely what the evidence shows. Stanley et al. (2017) had individuals consider counterfactual alternatives to remembered past events. They then asked them to judge the similarity between the remembered events and the alternatives as well as the plausibility of the counterfactual “events.” The results showed a strong correlation: the greater the perceived similarity, the more plausible the counterfactual alternative seemed to the participants. Indeed, the comparative similarity predicted most of the variance in plausibility ratings, beating out ease of imagination, frequency of rehearsal and so on. De Brigard et al. (2021) replicated this finding, demonstrating moreover that attending to the relevant similarities affects subjects’ judgments of plausibility. Results of this kind suggest, even if in a tentative way, that people engage in counterfactual episodic thought by manipulating “factual” models of past events (for a review, see De Brigard and Parikh, 2019).

Similar processes are at work in the imagination of possible future events. Research shows that the link between memory and episodic future thought (EFT) is particularly strong (D’Argembeau, 2015; Schacter et al., 2017). This is not only due to re-use of information about past events in EFT—in a recent study, participants reported that 90% of the details of imagined future events were associated with at least one memory (Anderson, 2012)—but also due to deeper similarities in processing (constructive retrieval, reliance on event schemas, goal-relevance etc.). In the simplest form of EFT, indeed, people simply “recast” a model of a past event to the future, a tactic useful for thinking about events with a shared fine structure (Addis et al., 2009). In more typical cases, the structural similarity is exploited in a flexible manner, by recombining elements from past events or manipulating relevant relations. The models, constructed upon retrieval, afford surrogative reasoning about the likely structure and dynamics of possible events. Admittedly, the investigation of the relationship between perceived plausibility of future events and their similarity to past events is in its infancy. Yet, there is some evidence that similarity, along with coherence with goals and personal characteristics, is of key importance (e.g., Ernst and D’Argembeau, 2017). It is in this context that mining the rich resources of mental model theory holds particular promise, with episodic modeling potentially employed in reasoning about the spatiotemporal, causal and conditional structure of event-types (Goldvarg and Johnson-Laird, 2001; Byrne and Johnson-Laird, 2009; Ragni and Knauff, 2013).

Mental models, hence, may be used to represent the *commonalities* among different situations (Johnson-Laird, 1983, 2006). I close by linking this idea to the phenomenon of general episodic memories, which represent multiple events of a specific type (Barsalou, 1988; Singer and Moffitt, (1991-1992); Berntsen, 1998; Renoult et al., 2012, 2016). In such memories, an episodic model is used to represent the shared structure of a series of events; a pattern of relations and activities, often situated in a specific spatiotemporal context. With the construction of a general model, information about such structure is integrated and made available for verbal report and surrogative reasoning. This process, the evidence suggests, is crucial not only for learning from the past, but also for learning about oneself (Conway, 2005, 2009; Renoult et al., 2012, 2016). In lieu of a summary, I offer an image from the prehistory of mental model theory: Peirce’s ((1931–1938/1958)) description of ideas as mental ‘‘composite photographs,’’ compiled from multiple experiences of relevantly similar events.^33^ This metaphor nicely captures the spirit of the proposal. A general event memory is a kind of mental composite photograph of a series of events.

## Conclusion

This paper offered a modeling account of episodic representation. On the account, the episodic system constructs manipulable mental models: representations that preserve the spatiotemporal structure and dynamics of events. The models are inference-supportive, allowing surrogative reasoning about the represented events. This analysis leaves a number of important questions open. These pertain to the incorporation of non*-*structural elements in episodic models, the characterization of different “dimensions” of episodic thought, such as specificity, subjectivity and tense (Mahr, 2020), as well as to the interaction between different systems in the production of complex states of remembering and imagination. A good understanding of the nature of episodic modeling is nevertheless necessary for exploring these difficult questions.

## Data availability statement

The original contributions presented in the study are included in the article/supplementary material, further inquiries can be directed to the corresponding author/s.

## Author contributions

The author confirms being the sole contributor of this work and has approved it for publication.

## Footnotes

1 The term can also refer to the output of that process, i.e., the representation generated by it. When necessary, I’ll mark the distinction between these two senses.

2 This narrow sense of “simulation” is familiar from the mindreading literature, where it is used to refer to the mental process of replicating (or “copying,” “re-enacting”) the mental processes/states of other agents (Currie and Ravenscroft, 2002; Goldman, 2006).

3 For Shanton and Goldman (2010), remembering involves the simulation of past *experiences/mental states*, not “external” events. Indeed, they see remembering as a kind of mindreading of one’s past self.

4 Cf. O’Brien and Opie (2004): “Nothing is more obvious than the fact that our minds are capable of representing features of the world that are not replicable in neural tissue. Moreover, even where the properties actually exemplified by neural tissue are concerned, it is most unlikely that these very often play a role in representing those self-same properties in the world.” (p. 9).

5 I return to this issue in section “Episodic simulation: Polishing the common core.”

6 Structural representations are often called “iconic representations.” Indeed, that is the term preferred by the leading proponent of mental model theory (e.g., Johnson-Laird, 2006, Ch. 2). While these terms can be used interchangeably, I prefer “structural.” This is partly a result of a growing trend in philosophy and cognitive science (Shagrir, 2012; Shea, 2014; Gładziejewski and Miłkowski, 2017). But it is also because the term “iconic” tends to be associated with *mental imagery*, an association I want to avoid.

7 I return to this issue in “Modeling and surrogative reasoning.”

8 The second-order resemblance should be contrasted with *first*-order (physical) resemblance between objects/domains and their properties (e.g., representing space with space) (see Shepard and Chipman, 1970; Cummins, 1996; O’Brien and Opie, 2004).

9 This is an adaptation of Peirce’s analysis, borrowed from Von Eckardt (1993).

10 Cf. Johnson-Laird (2006): “The moral is that representations are [structural] because of the way in which the interpretative system uses them. A representation in itself does not convey its interpretation” (p. 33). While Johnson-Laird here uses “representation” for the mapping relation subject to interpretation, I reserve “representation” for the *interpreted/used* resemblance relation. Despite this passage, I take Johnson-Laird to be on board with the latter use.

11 Theorists that insist on defining iconicity in terms of Fodor’s “part principle” may want to stress that mental models are not *purely* iconic in the way pictures are (e.g., Quilty-Dunn and Mandelbaum, 2019). This is undoubtedly correct; see section “Episodic representation: What the thesis is not.”

12 For the importance of locality in structural representations (see Kuipers, 2007; Robins et al., 2022).

13 The entorhinal cortex (Brodmann area 28) is an area located in the medial temporal lobe, forming a nodal point between the hippocampal formation and a number of multimodal association areas of the cortex (for its anatomical organization, see Canto et al., 2008).

14 Importantly, this does *not* entail that motor planning and navigation is entirely (and exclusively) dependent on representations of this kind. On the contrary, it is likely that complex dynamical systems can/will employ a variety of strategies and solutions for navigating even simple trajectories (for a good discussion see Ekstrom et al., 2020).

15 Hippocampal time cells have been observed in rodents, monkeys and, most recently, in humans (Umbach et al., 2020).

16 Bellmund et al. (2018) define properties as convex regions of cognitive spaces, where convexity entails that “for all points x and y in the region, the points between x and y also fall into the region” (p. 1). Complex concepts span multiple different domains and are defined as *sets* of convex regions in multiple domains (see also Balkenius and Gärdenfors, 2016).

17 Behrens et al. (2018) offer a related proposal of comparative ambition. The mapping of spatial context by the hippocampal-entorhinal circuit, they argue, is only one instance of a general coding mechanism, capable of organizing knowledge of all kinds. Bringing in ideas from reinforcement learning, they demonstrate that grid-like representations can encode knowledge about the reward structure of complex (non-spatial) tasks, i.e., about the relationships between different states in a task state-space. They show that there is a set of vectors that afford the computation of distance between any pairs of states (eliminating the need for “step-by-step” simulation). These “eigenvectors” have been found to be important components of place and grid cell activity (Stachenfeld et al., 2017).

18 This framing is borrowed from Ekstrom and Ranganath (2018). Note that (a) the boundaries of the represented events can be manipulated, and (b) events can subsequently be integrated into larger representative structures, e.g., representations of lifetime periods (Conway, 2005). These larger structures have different cognitive signatures, however.

19 The evidence that *other* animals automatically segment their activity/experience into discrete events is slowly accumulating (for important recent studies, see Gupta et al., 2012; Sun et al., 2020).

20 The representation of spatiotemporal context is *widely* considered to be a distinguishing characteristic of episodic memory. For many, indeed, this is a direct consequence of the fact that episodic memory represents past events *as experienced.* Russell and Hanna (2012) provide a Kantian framing: “For Kant, spatial and temporal content is not something derived from experience, or something that it is possible for experience to be enjoyed without, but it is that on which experience depends as a necessary framework. Just as experience must involve an experiencer’s spatial perspective on objects and events, so to must re-experience” (p. 32–33).

21 There has been a lot of discussion about the proper way to understand the notion of “allocentric representation.” While the nuances are both interesting and relevant, there are beyond my scope here. For a classic proposal, see O’Keefe and Nadel’s *The Hippocampus as a Cognitive Map* (1978). For a philosophical treatment and measured criticism of the proposal (see Campbell, 1995). For an analysis of the ways in which the notion is used in contemporary rodent neuroscience (see Wang et al., 2020).

22 Arzy and Schacter (2019), in a recent essay, suggest that the allocentric-egocentric interplay in spatial coding can also be linked to the coding of perspective in memory. This is an interesting idea, but we should proceed with care. Thus, it may thus be tempting to assume that first-person “field” memories (Nigro and Neisser, 1983) involve the construction and manipulation of egocentric representations (with “observer” memories linked to allocentric representations). This may end up being the case, but it is not something that can be established solely on phenomenological grounds. The complexity of the system makes it likely that systematic allocentric-egocentric transformations play a role in both field and observer memories (Rolls, 2020). What matters for our purposes, in any case, is the structure-preserving mapping of an event’s spatial context, which is independent of the *experienced* perspective (cf. Johnson-Laird, 2006, Ch. 2).

23 Cf. (2017, p. 346, emphasis original): “Interventions on the vehicle’s structure change the success *only insofar as they change the degree of similarity between the vehicle and the target*. They increase success if they increase the structural fit between the vehicle and the target. They decrease success only if they decrease the structural fit. And they do not change the success if they do not bring about any change in the structural fit. In any case, what the success depends on is not just the vehicle, but also structural similarity.”

24 An interesting feature of the replay mechanism is that sequences are replayed at *compressed timescales* (Buzsáki and Moser, 2013). It is tempting to speculate that this feature, combined with the partiality of the structure-preserving mappings, plays a role in producing the experience of “reliving” events in a temporally condensed form (as “trajectories-within-snapshots”; Altmann and Ekves, 2019).

25 I am grateful to an anonymous referee for bringing my attention to the work of Faber and Gennari.

26 These results motivated the development of the *scene construction theory* (Hassabis et al., 2007; Hassabis and Maguire, 2009). Here, I propose that the so-called “scene construction” is best understood as the construction of structural mental models of temporally extended events (see section “Episodic simulation: Polishing the common core”).

27 A strong version of this thesis holds that representations produced at *all* stages of stimulus processing can be registered in memory and potentially be made available for recall (see, e.g., Lansdale, 2005).

28 Because this entailment doesn’t hold, one cannot infer the content of systemic representations *from* the (introspected) content of episodic memory experiences. See Pan (2022) for an excellent treatment of this issue.

29 I am grateful to an anonymous referee for urging me to clarify this commitment.

30 Thanks to a referee for prompting me to add this qualification.

31 You may, for example, think of this information as being “globally broadcast” (Baars, 1988). However, you need not be committed to any strong view about the architecture of cognition (or consciousness) to appreciate the relevance of representational extraction.

32 This is primarily because of working memory limitations, hindering thinkers from holding multiple fully fleshed out models of situations in mind (Byrne, 2002).

33 “Take, for instance, ‘it rains.’ Here the icon is the mental composite photograph of all the rainy days the thinker has experienced” (2.438).
